# Metabolic reprogramming as a therapeutic target for modulating the Th17/Treg balance in autoimmune diseases: a comprehensive review

**DOI:** 10.3389/fimmu.2025.1687755

**Published:** 2025-12-16

**Authors:** Yuehong Hu, Qihan Zhao, Haoran Dai, Yadi Wu, Xinyue Tang, Naiqian Zhang, Hanxue Jiang, Hongliang Rui, Baoli Liu

**Affiliations:** 1 Center of Nephrology and Rheumatology, Beijing Hospital of Traditional Chinese Medicine, Capital Medical University, Beijing, China; 2 Laboratory for Clinical Medicine, Capital Medical University, Beijing, China; 3 Beijing Institute of Chinese Medicine, Beijing, China

**Keywords:** immunometabolism, Th17/Treg balance, autoimmune diseases, therapeutic target, mTOR

## Abstract

The dynamic balance between T helper 17 (Th17) cells and regulatory T (Treg) is the cornerstone of immune homeostasis. Disruption of this equilibrium is closely associated with various autoimmune diseases, including rheumatoid arthritis (RA), multiple sclerosis (MS), and inflammatory bowel disease (IBD). Studies have revealed that metabolic reprogramming, mediated by key metabolic enzymes (including mTOR, HIF-1α, and AMPK) and pathways (such as glycolysis and lipid metabolism), acts as a major regulator of Th17/Treg differentiation and function owing to their distinct metabolic profiles. Metabolic dysregulation may exacerbate immune imbalance by altering the cellular differentiation trajectories and functional states. Although targeting metabolic pathways shows therapeutic promise, current intervention strategies face challenges in terms of specificity and safety. This review systematically combs the mechanisms by which metabolic reprogramming influences the differentiation and function of Th17/Treg cells, as well as the metabolic changes in immune cells of inflammation-related autoimmune diseases. It outlines the progress of the latest metabolism-targeted strategies and focuses on discussing the challenges and prospects regarding the specificity and safety of metabolic interventions.

## 1 Introduction

The dynamic balance among CD4^+^ T cell subsets is essential for immune homeostasis, and its disruption is a hallmark of autoimmune pathology. A critical axis in this balance is between pro-inflammatory T helper 17 (Th17) cells and anti-inflammatory regulatory T (Treg) cells (1, 2). Recent advances in immunometabolism have revealed that the distinct functional identities of these subsets are underpinned by divergent metabolic programs: Th17 cells predominantly utilize aerobic glycolysis and fatty acid synthesis (FAS) to sustain their pro-inflammatory phenotype, whereas Treg cells rely on oxidative phosphorylation (OXPHOS) and fatty acid oxidation (FAO) to maintain their suppressive function. These metabolic pathways act as crucial checkpoints that direct T cell fate (1, 3, 4).

**Figure 1:**
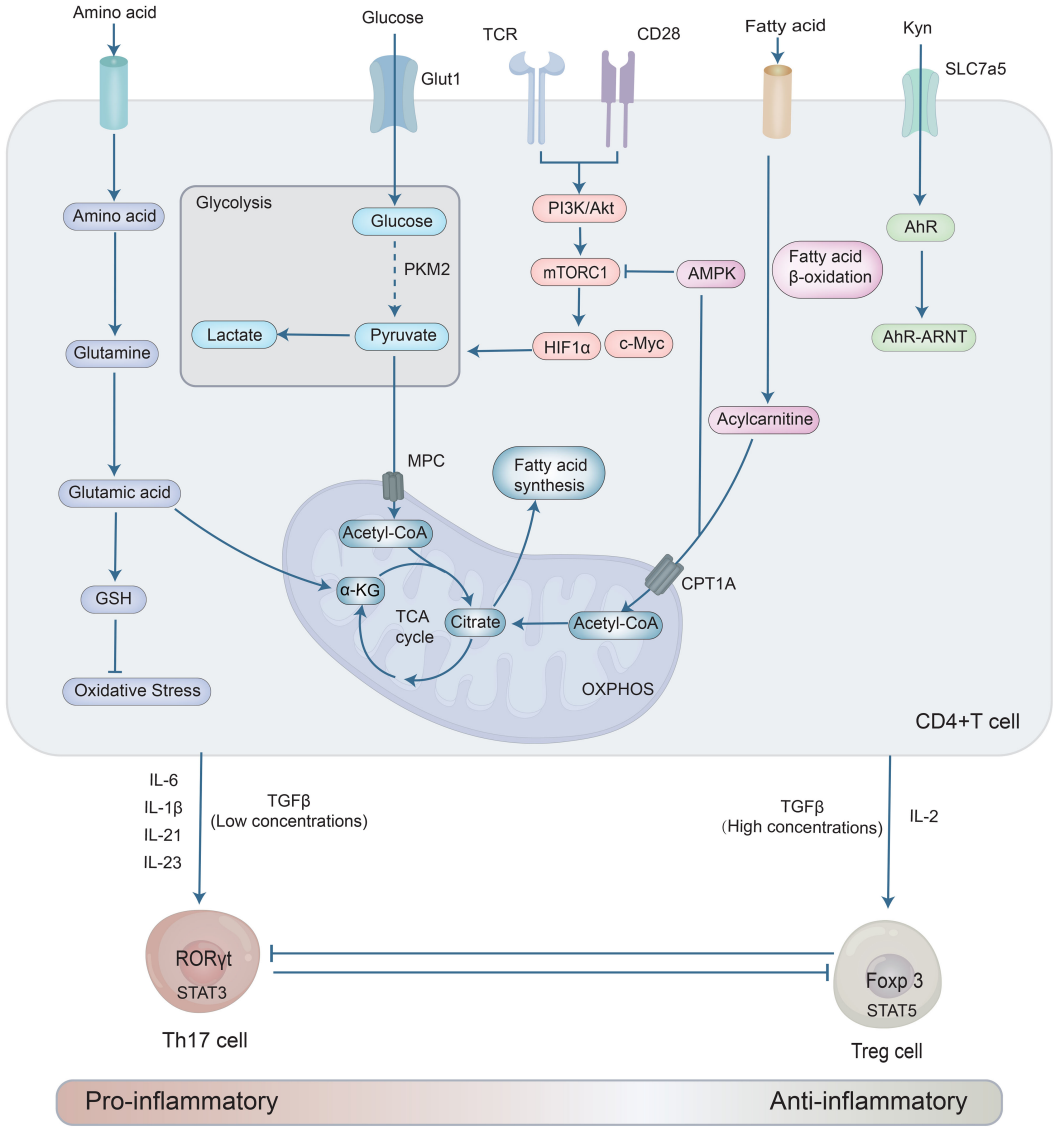
Metabolic and signaling pathways regulate the differentiation and functional balance between pro-inflammatory Th17 and anti-inflammatory Treg cells. In this process, naive CD4^+^ T cells differentiate under the influence of cytokines and metabolic reprogramming, whereby the Th17 pathway is driven by glycolysis and fatty acid synthesis via the PI3K/Akt/mTORC1 axis, while Treg development depends on fatty acid oxidation and oxidative phosphorylation activated by AMPK. Key transcription factors RORγt (for Th17) and Foxp3 (for Treg) mutually inhibit each other, establishing an antagonistic cell-fate circuit.

However, many existing reviews have primarily catalogued these metabolic differences in isolation. The novelty of this review lies in its integrative approach, moving beyond a mere description of disparate pathways to synthesize a coherent framework that connects key metabolic nodes—such as the mammalian target of rapamycin (mTOR)-hypoxia-inducible factor 1-alpha (HIF-1α)-glycolysis axis, AMP-activated protein kinase (AMPK)-FAO signaling—into a dynamic immunometabolic network (**Figure 1**). We focus specifically on how the dysregulation of this network drives pathogenic Th17/Treg imbalance across diverse autoimmune diseases, including rheumatoid arthritis (RA), multiple sclerosis (MS), inflammatory bowel disease (IBD), and systemic lupus erythematosus (SLE). Although current inhibitors (such metformin and rapamycin) can correct the Th17/Treg imbalance, they are likely to impact non-target cells and result in metabolic issues. Thus, accurate therapy is still a critical issue that needs to be resolved.

As a result, we systematically explore the potential of targeting these specific metabolic pathways to achieve a more precise restoration of immune balance. By framing metabolic reprogramming as a network-level target, this review offers a novel perspective on overcoming the specificity challenges that have hindered the clinical application of immunometabolism research.

## 2 Characteristics of Th17 cells and Treg cells

Th17 and Treg cells, despite their shared origin in naïve CD4^+^ T cells, undergo divergent differentiation fates dictated by antagonistic transcriptional and cytokine networks. Their commitment is primarily governed by the mutually inhibitory master regulators retinoic acid-related orphan receptor gamma-t (RORγt) (driving the Th17 lineage) and forkhead box P3 (Foxp3) (orchestrating the Treg program), within cytokine milieus that favor either pro-inflammatory (e.g., IL-6, IL-23) or anti-inflammatory (e.g., TGF-β, IL-2) outcomes (**Figure 1**). Consequently, a precise understanding of the signals that govern these cell fates is fundamental to developing therapies that can recalibrate the Th17/Treg balance in disease.

### 2.1 The antagonistic relationship between Th17/Treg during differentiation pathways

#### 2.1.1 Transcription factor regulation

A vital mechanism in the control of immunological homeostasis is the mutual antagonistic relationship between RORγt and Foxp3 (**Figure 1**), which is a crucial intrinsic factor affecting the differentiation pathways of Th17 and Treg cells.

As the main transcription factor of Th17 cell, RORγt collaborates with other transcription factors, such as RORα, IRF4, and Runx1, to synergistically stimulate Th17 cell growth and IL-17 release (5). On the other hand, Foxp3 inhibits the transcriptional activity of RORγt and Runx1, which in turn suppresses the expression of IL-17 (6). In addition to fighting for acetylation sites on Foxp3 and encouraging its proteasomal destruction, transcriptional co-activator with PDZ-binding motif (TAZ) acts as a co-activator of RORγt, increasing its transcriptional efficacy (7).

Foxp3 interacts with several transcription factors, such as STAT5, NFAT, and BACH2, to influence the Treg cells' development, stability, and epigenetic modifications. There is a direct correlation between its expression level and Tregs' ability to inhibit (8, 9). Furthermore, Foxp3 regulates gene expression by interacting with several transcription factors, including NFAT and AML1/Runx1, and upregulating molecules like CD25, cytotoxic T lymphocyte-associated antigen-4 (CTLA-4), and glucocorticoid-induced TNFR-related protein (GITR) while decreasing pro-inflammatory cytokines, such as IL-17 (10).

A critical layer of complexity arises from the functional heterogeneity and phenotypic plasticity within the Treg compartment. While traditionally viewed as a stable lineage committed to immunosuppression, emerging evidence indicates that certain Treg subsets can acquire effector-like characteristics under inflammatory conditions, including the production of pro-inflammatory cytokines. Notably, This type of Treg cell co-expressing Foxp3 and RORγt (Th17-like Tregs) has been identified in humans and mice (11, 12). Under inflammatory conditions, Th17-like Tregs may undergo phenotypic conversion, characterized by diminished Foxp3 expression and acquisition of a pro-inflammatory, Th17-like phenotype accompanied by IL-17 production, thereby potentially contributing to autoimmune pathology (13, 14). However, the stability and functional relevance of these Th17-like Tregs *in vivo* remain subjects of ongoing debate. While some studies suggest that such cells may retain certain regulatory functions, others indicate that they can adopt a pathogenic role (15, 16). At the molecular level, Foxp3 has been shown to interact with RORγt via its exon 2 domain under homeostatic conditions, thereby suppressing RORγt-mediated transcriptional activity and IL-17A production (17). This repression can be overcome in the presence of inflammatory cytokines such as IL-6, IL-21, and IL-23, which disrupt Foxp3–RORγt interaction and favor Th17 differentiation (17). Consequently, these functional ambiguities may reflect the inherently unstable identity of Th17-like Tregs, whose regulatory or pathogenic output is critically dependent on the surrounding inflammatory context.

#### 2.1.2 Regulation of cytokine signaling

Th17 and Treg cells' differentiation paths are determined by the cytokine microenvironment, an extrinsic factor (18), with TGF-β and other factors being essential for their lineage commitment. Although its mechanistic actions in different microenvironmental situations, TGF-β have selective effects on these immune subsets' lineage commitment (19).

The induction of non-pathogenic Th17 cells under homeostatic conditions, which are characterized by the expression of genes like Il10, requires low concentrations of TGF-β in conjunction with IL-6. These cells also help mucosal immune surveillance by secreting IL-10 and minimal IL-17 (18). Conversely, TGF-β (or IL-1β), IL-6, and IL-23 stimulate the development of pathogenic Th17 cells (20, 21). These cells have high levels of pro-inflammatory genes such as Csf2, Ifng and Il23r, and they secrete a lot of granulocyte-macrophage colony-stimulating factor (GM-CSF) and IL-17A, which exacerbates tissue inflammation (22–25). The ability of IL-23 to inhibit IL-10 production and work in concert with IL-1β to activate STAT3 and RORγt transcriptional programs is what causes this pathogenic shift, which helps Th17 cells change into a pathogenic phenotype (20, 26–30). In addition, activated STAT3 can suppress Foxp3 transcriptional activity, which in turn inhibits Treg cell differentiation (31). High concentrations of TGF-β block the transcriptional activation of RORγt and promote the development of Treg cells by suppressing the production of IL-23R and facilitating Foxp3's direct binding to RORγt (17, 32–34). It promotes Treg cell development by activating the Small mothers against decapentaplegic2 (SMAD2) and SMAD3 signaling pathways, which in turn stimulates Foxp3 expression when TGF-β is present alone (35, 36).

According to *in vitro* research, IL-2 and TGF-β work together to decrease the production of IL-17 and stimulate STAT5 phosphorylation, which accelerates the development of naïve CD4^+^ T cells into induced regulatory T cells (iTregs) (37). However, these iTregs often exhibit instability, with a tendency to lose Foxp3 expression and convert to Foxp3^-^ Effector T cells (Teff) cells. This instability may result from inadequate demethylation of conserved non-coding sequence regions in the Foxp3 locus, known as the Treg-specific demethylation region (TSDR), which is fully demethylated in thymus-derived Tregs (tTregs) but often methylated in iTregs (38). Foxp3 stability in iTregs can be greatly increased by optimizing culture conditions, especially by adding epigenetic modifiers such vitamin C (39, 40). Moreover, antigen-specific iTregs demonstrate substantially improved immunoregulatory activity and Foxp3 expression stability (41). Nevertheless, a critical caveat is that many findings on iTreg stability come from *in vitro* systems, and their physiological relevance, particularly in human disease, requires further validation. Notably, while mouse iTregs can be highly suppressive *in vivo*, human iTregs generated under similar conditions often fail to acquire full regulatory function, highlighting important species-specific differences (38). These differences underscore the necessity of cautious extrapolation from mouse models to human immunobiology and highlight the need for further validation in physiologically relevant settings.

### 2.2 The functional antagonism between Th17 cells and Treg cells

The functional antagonism between Th17 cells and Treg cells primarily manifests in their reciprocal interactions and regulatory mechanisms within the immune system. Th17 cells predominantly mediate host defense under physiological conditions by protecting against external pathogens. The functional antagonism between Th17 cells and Treg cells primarily manifests in their reciprocal interactions and regulatory mechanisms within the immune system (18, 42). As an example, Th17 cells in RA secrete pro-inflammatory cytokines including IL-17 and IL-23, which promote the growth of synovial fibroblasts and cause synovial hypertrophy (43). These cytokines also increase the production of matrix metalloproteinases (MMPs) and receptor activator of nuclear factor kappa-B ligand (RANKL), which leads to osteoclastogenesis and osteoclast activation, which deteriorates bone and cartilage in joints (44, 45).

Treg cells mainly fall into four categories and use direct or indirect interaction to carry out immunosuppressive actions: 1) Cytokine-mediated suppression: TGF-β, IL-10, and IL-35 induce Treg development, prevent antigen-presenting cell (APC) maturation, and mediate bystander suppression (46). 2) Contact-dependent suppression, such as the inhibition of conventional T cell (Tconv) activity by the surface molecule CTLA-4 competing with CD80/CD86 on APCs, inhibiting co-stimulatory signals, and upregulating PD-L1 expression (47). 3) Metabolic regulation: Foxp3+ Treg differentiation and suppressive ability are enhanced by the catalysis of tryptophan breakdown to kynurenine by Indoleamine 2,3-dioxygenase (IDO) (48). 4) Transcriptional regulation: Differential activation of STAT proteins gives Treg subsets particular suppressive functions. For example, Tregs with activated STAT3 can suppress Th17 responses (intestinal inflammation results from its deficiency), and T-bet expression can further suppress T helper 1 (Th1) and CD8+ T cells (8, 49).

## 3 Metabolic programs governing the Th17/Treg balance

Metabolic reprogramming critically controls Th17 and Treg cell fate. This regulation involves microenvironmental nutrients and intrinsic metabolic pathways. Extrinsically, dietary long-chain fatty acids (LCFAs) promote Th17 cells and exacerbate the pathogenesis of experimental autoimmune encephalomyelitis (EAE) (50). Conversely, short-chain fatty acids (SCFAs) enhance Treg differentiation and suppress Th17 activities, alleviating EAE and IBD (51–53). Amino acid metabolism also plays a key role. Tryptophan catabolites activate the aryl hydrocarbon receptor (AhR) to promote Treg cell polarization (54). Conversely, Leucine promotes Th17 cell differentiation and IL-17 production through SLC7a5-mediated influx, and subsequent activation of the mTOR complex 1 (mTORC1)-HIF-1α signaling axis (55). Intrinsically, HIF-1α drives Th17 cell development. It boosts glycolysis, activates RORγt, and degrades Foxp3 (56, 57). Thus, beyond cytokines and transcription factors, integrated metabolic pathways—including glycolysis, fatty acid and amino acid metabolism—are fundamental regulators of the Th17/Treg balance and immune homeostasis (**Figure 1**).

### 3.1 Glucose-centric regulation

Glycolytic flux is a hallmark of effector T cell activation, supporting biosynthetic demands and redox homeostasis, and influencing the Th17/Treg balance. Key nodes include:

#### 3.1.1 GLUT1/3: mediated glucose uptake

Glucose transporter1 (GLUT1) expression is modest in resting T cells, but co-stimulation through T cell receptor (TCR) and CD28 quickly triggers the phosphoinositide 3-kinase (PI3K)/Protein Kinase B (Akt)/mTOR signaling cascade, which results in GLUT1 transcriptional upregulation and membrane translocation (58, 59). To address the metabolic needs of T cells, this leads to a 10–20 fold increase in glucose uptake (60, 61). Th17 cells co-express GLUT1 and GLUT3 to sustain high glycolytic activity, whereas Treg cells exhibit suppressed glycolysis via Foxp3-mediated inhibition of Akt/mTOR signaling, favoring OXPHOS (62–65). Within pathological settings, the critical link between metabolic reprogramming and immunocyte function becomes most apparent. Evidence from colitis models demonstrates that GLUT1 ablation preferentially dampens Th17 cell expansion, while largely preserving the regulatory capacity of Treg cells (66), underscoring the potential for precise immunometabolic targeting.

#### 3.1.2 PKM2: driven metabolic rewiring

Pyruvate kinase is the rate-limiting enzyme that catalyzes the conversion of phosphoenolpyruvate (PEP) to pyruvate, serves as a critical metabolic and immunological node in T cell fate determination. Its function is intricately related by its oligomeric state: the ​​tetrameric form​​ localizes to the cytoplasm and exhibits high pyruvate kinase activity, channeling glycolytic flux, whereas the ​​dimeric form​​ translocates to the nucleus upon TCR activation, where it functions as a protein kinase and transcriptional coactivator (67). This activates the production of HIF-1α, mTORC1, and Myc, which are crucial metabolic support systems for Th17 cell development. In particular, Th17 cell development is promoted by the buildup of glycolytic intermediates, which further activates transcription factors including RORγt, Runx1, and Irf4 (67, 68). The allosteric activator TEPP-46 inhibits the expression of glycolytic genes by inducing pyruvate kinase M2 (PKM2) tetramerization and blocking its nuclear translocation. This intervention significantly suppresses the differentiation and inflammatory cytokine production (e.g., IL-17A) of pathogenic Th17 cells, while concurrently promoting the generation of Treg cells (67). The therapeutic potential of this mechanism is evidenced by the efficacy of TEPP-46 and the natural compound shikonin in ameliorating disease in EAE and colitis models (68–70). Interestingly, Cellular metabolic plasticity is evidenced by compensatory upregulation of PKM1 expression in response to PKM2 deficiency, which can partially restore glycolytic flux (71). Conversely, the transcription factor c-Myc can promote a splicing switch from PKM1 to PKM2, thereby augmenting glycolytic capacity to support T cell proliferation (72). Collectively, these findings establish PKM2 as a pivotal metabolic checkpoint, whose allosteric regulation offers a compelling strategy for immunomodulation by skewing the balance away from pro-inflammatory Th17 responses and toward Treg cell functions.

#### 3.1.3 mTOR/AMPK/HIF-1α axis: an integrated metabolic checkpoint

The mTOR–AMPK–HIF-1α axis serves as a central signaling hub that integrates nutrient availability, energy status, and immune signals to direct T cell differentiation (73). mTOR exists in two structurally and functionally distinct complexes, mTORC1 and mTORC2, which are activated downstream of PI3K/Akt signaling upon TCR and CD28 costimulation (4, 74). mTORC1 promotes anabolic metabolism by enhancing glycolysis, lipid synthesis, and amino acid metabolism, thereby driving Th17 cell differentiation through transcriptional upregulation of sterol regulatory element-binding proteins (SREBPs), HIF-1α, and c-Myc (75). Conversely, mTORC2 supports Treg cell development by regulating FAO and moderating glycolytic flux (76). While mTORC1 deficiency impairs Th17 cell differentiation, the concurrent inhibition of both mTORC1 and mTORC2 signaling is required for the potent induction of Treg cell differentiation (77). However, The function of mTOR in controlling Treg cells is still up for debate, though; while extended exposure to rapamycin seems to encourage Treg proliferation, short-term exposure suppresses mTOR signaling and increases Foxp3 (78). Subsequent investigation shows that mTOR signaling dynamically controls Treg cell migration and stability in inflammatory settings (79). Thus, cellular metabolic pathways and immunological responses (such as the production of important transcription factors) are closely regulated in both directions, either preserving immune homeostasis or aiding in the pathophysiology of illness.

AMPK acts as an energy-sensing counterbalance to mTOR (80). Activated under low ATP conditions, AMPK phosphorylates TSC2 and Raptor to inhibit mTORC1, thereby promoting catabolic pathways such as FAO and mitochondrial biogenesis (81). This metabolic shift favors Treg differentiation and suppresses Th17 development. Notably, AMPK activation by metformin or sustained TGF-β signaling enhances Treg generation and facilitates Th17-to-Treg conversion, highlighting its role in immune tolerance (82, 83). However, AMPK’s effects are context-dependent: under nutrient-replete conditions, AMPK-deficient T cells maintain normal responses, but during glucose restriction or metabolic stress, their adaptability is severely impaired (84).

HIF-1α, stabilized by mTORC1 signaling and hypoxic microenvironments, reinforces glycolytic metabolism and Th17 commitment (85). It directly transactivates RORγt while promoting Foxp3 degradation via the ubiquitin-proteasome pathway (86). Paradoxically, under prolonged hypoxia, HIF-1α can also support Treg stability and function, indicating microenvironment-dependent duality (87–89). Genetic depletion of HIF-1α in T cells enhances oxidative metabolism and Treg output while suppressing Th17 responses, confirming its pivotal role in metabolic reprogramming (57).

The interplay between these regulators creates a dynamic checkpoint: mTORC1 and HIF-1α synergize to propel effector T cell responses under nutrient-rich conditions, whereas AMPK activation restrains anabolic metabolism to favor regulatory phenotypes during energy stress. This axis not only determines Th17/Treg balance but also offers therapeutic targets for autoimmune and inflammatory diseases.

### 3.2 Bidirectional regulation of lipid metabolism

Lipid metabolism serves as a critical bidirectional regulator of CD4^+^ T cell differentiation, dynamically shaping the balance between pro-inflammatory Th17 cells and immunoregulatory Treg cells through integrated control of extracellular lipid uptake, intracellular synthesis, and FAO (90, 91). This metabolic network allows T cells to adapt to microenvironmental cues, with distinct lipid utilization patterns favoring either effector or regulatory phenotypes (92).

#### 3.2.1 Uptake and signaling of exogenous lipids

Extracellular lipids, particularly fatty acids of varying chain lengths, exert selective effects on T cell fate. SCFAs such as butyrate—produced by gut microbiota—promote Treg differentiation and function via G protein-coupled receptor (GPR43/FFAR2) signaling, enhancing Foxp3 and IL-10 expression (93). SCFA deficiency exacerbates inflammation in colitis and arthritis models (93, 94). Oleic acid strengthens the suppressive function of Treg cells via enhancing FAO-driven OXPHOS, which in turn activates STAT5 signaling (95). It is important to note that in this context, microbiota-derived immunomodulatory metabolites refer primarily to SCFAs and bile acid derivatives, rather than LCFAs. In contrast, dietary ​​LCFAs drive naïve T cell polarization toward Th17 cells through p38 MAPK signaling, aggravating pathology in experimental autoimmune encephalomyelitis (50). This finding suggests that dietary LCFAs restriction strategies may serve as adjunctive therapies for autoimmune diseases. Additionally, cholesterol metabolites like ​​bile acids​​ suppress Th17 activity while promoting colonic Treg development, illustrating how systemic lipid sources can locally influence immune homeostasis (96–98).

#### 3.2.2 Endogenous fatty acid synthesis and oxidation

T cell subsets display divergent reliance on *de novo* lipogenesis versus FAO. Th17 cells depend on FAS for membrane biosynthesis and inflammatory signaling, requiring enzymes such as acetyl-CoA carboxylase 1 (ACC1) and fatty acid synthase (FASN) (99). ACC1 deficiency impairs RORγt binding to the IL-17 promoter, reducing Th17 output while favoring Treg generation (100, 101). Conversely, Treg cells maintain functional fitness primarily through ​​mitochondrial FAO​​, which supports OXPHOS and sustains their suppressive capacity (102). The AMPK-ACC-CPT1 axis acts as a key regulatory node: AMPK activation phosphorylates ACC, reducing malonyl-CoA levels and relieving inhibition of CPT1 to enhance FAO and Treg differentiation (103). Pharmacologic activation of this pathway by metformin amplifies Treg populations (90, 104), while glycolysis inhibition with 2-deoxyglucose (2-DG) shifts metabolism toward FAO, thereby promoting a phenotypic switch from Th17 to Treg cells (82).

FAO-driven OXPHOS is necessary for Treg cells to continue their suppressive role (105). Notably, Foxp3 improves Treg adaptability to lipotoxic conditions by controlling lipid absorption and metabolic enzyme expression (106), without compromising their glycolysis function (107), TGF-β signaling encourages the differentiation of naïve T cells into Tregs by upregulating CPT1 (108). Interestingly, Treg function in mice lacking the CPT1 inhibitor etomoxir is not severely impacted, despite the fact that it can impede Treg formation (109), indicating the presence of compensating regulatory mechanisms for FAO.

In summary, lipid metabolism orchestrates Th17/Treg balance through multiple interconnected mechanisms: extracellular lipids provide environment-dependent signals, endogenous synthesis and oxidation pathways fuel functionally distinct metabolic states. Targeting these pathways—such as by restricting dietary LCFAs or activating AMPK/CPT1—offers promising strategies for immunomodulation in autoimmune and inflammatory diseases.

### 3.3 Amino acid metabolism-specific pathway

Amino acids serve not only as fundamental building blocks for protein and nucleotide synthesis but also as critical regulators of T cell differentiation and function through metabolic reprogramming (110). Distinct amino acid utilization patterns dynamically shape the balance between Th17 cells and Treg cells, particularly in response to microenvironmental cues such as inflammation, autoimmunity (73). This section examines the specific roles of glutamine, tryptophan, and leucine in directing immune cell fate through interconnected metabolic and signaling pathways.

#### 3.3.1 Glutamine metabolism and immunometabolic switching

Glutamine, a conditionally essential amino acid, plays a central role in T cell metabolic reprogramming (111). Through glutaminolysis, glutamine is converted to glutamate by glutaminase and subsequently to α-ketoglutarate (α-KG), which enters the tricarboxylic acid (TCA) cycle. α-KG acts as a cofactor for epigenetic enzymes such as TET dioxygenases and Jumonji histone demethylases, directly influencing the DNA methylation status of the Foxp3 locus (112). Restricting glutamine availability or inhibiting α-KG production promotes Foxp3 expression, facilitating the conversion of Th17 cells into Treg-like cells and enhancing Treg differentiation while suppressing Th1/Th17 polarization (113–115). Additionally, glutamine-derived glutamate stimulates the synthesis of glutathione (GSH), which preserves redox homeostasis (116, 117). Altered GSH levels serve as a redox checkpoint: GSH deficiency impairs RORγt-mediated Th17 differentiation while stabilizing Foxp3 to reinforce Treg function (118, 119).

Therapeutic strategies targeting glutamine metabolism—such as the antagonist DON, combined with glycolytic inhibitor 2-DG or metformin—can suppress effector T cell responses, though the effects of glutamate supplementation may vary depending on the pathological context, necessitating tailored approaches (120, 121).

#### 3.3.2 Tryptophan catabolism and immune tolerance

Tryptophan metabolism, primarily via the kynurenine pathway catalyzed by IDO1, establishes an immunosuppressive microenvironment (122). IDO1, which is widely expressed by tumor cells and APCs, controls T cell activity by reducing tryptophan and building up kynurenine metabolites in the microenvironment (122, 123). Tryptophan deficiency activates the GCN2 kinase-mediated integrated stress response, inhibiting T cell proliferation, while kynurenines promote AhR-driven Foxp3+ Treg differentiation and suppress Th17 polarization (54, 124). Although preclinical studies in colon cancer models show that IDO1 inhibition can enhance T cell infiltration when combined with anti–CTLA-4 therapy (125, 126), clinical trials of IDO1 monotherapy have demonstrated limited efficacy (127), reflecting tumor-specific metabolic adaptations and compensatory mechanisms. Notably, T cells uptake kynurenine via the SLC7a5 transporter (128), and recent evidence suggests kynurenine may induce T cell death by enhancing fatty acid oxidation, further contributing to immune suppression (129). The complexity of tryptophan metabolism, including its indirect effects on Treg abundance *in vivo*, underscores the challenge of targeting this pathway for immunotherapy.

#### 3.3.3 Leucine sensing and mTORC1-mediated metabolic checkpoint

Leucine, as a key activator of mTORC1 signaling, can regulate T cell metabolic checkpoint through the Sestrin 2 sensor. Studies have found that deficiencies in the leucine transporter SLC7a5 lead to insufficient leucine uptake, which limits T cell activation and Th17 cell differentiation by suppressing the expression of c-Myc and the activity of mTORC1 (55, 130). This disruption prevents metabolic reprogramming toward an effector phenotype, inhibiting Teff cell differentiation while potentially favoring Treg generation. Leucine-mediated mTORC1 signaling thus integrates nutrient availability with immune activation, highlighting its role in balancing inflammatory and regulatory responses.

Therefore, the metabolic pathways of glutamine, tryptophan, and leucine converge on key regulatory nodes such as mTOR, AhR, and epigenetic modifiers, creating a network that dynamically controls Th17/Treg equilibrium. The interplay between these amino acids illustrates the complexity of immunometabolic regulation, for example, leucine-driven mTOR activation and glutamine-derived α-KG–mediated epigenetic remodeling.

In summary, the metabolic programs governing Th17 and Treg cell differentiation—glucose utilization, lipid metabolism, and amino acid processing—operate as an integrated network rather than isolated pathways, dynamically interacting through central checkpoint nodes such as the mTOR-AMPK-HIF-1α axis. Therapeutic interventions must account for this crosstalk, since targeting a single pathway often proves inadequate due to compensatory adaptive mechanisms. Combining metabolic inhibitors with immunotherapies—such as glycolytic inhibitors with checkpoint modulators—represents a promising strategy to reestablish immune balance in autoimmune and inflammatory diseases.

## 4 Immunometabolic dysregulation in autoimmune diseases

**Figure 2:**
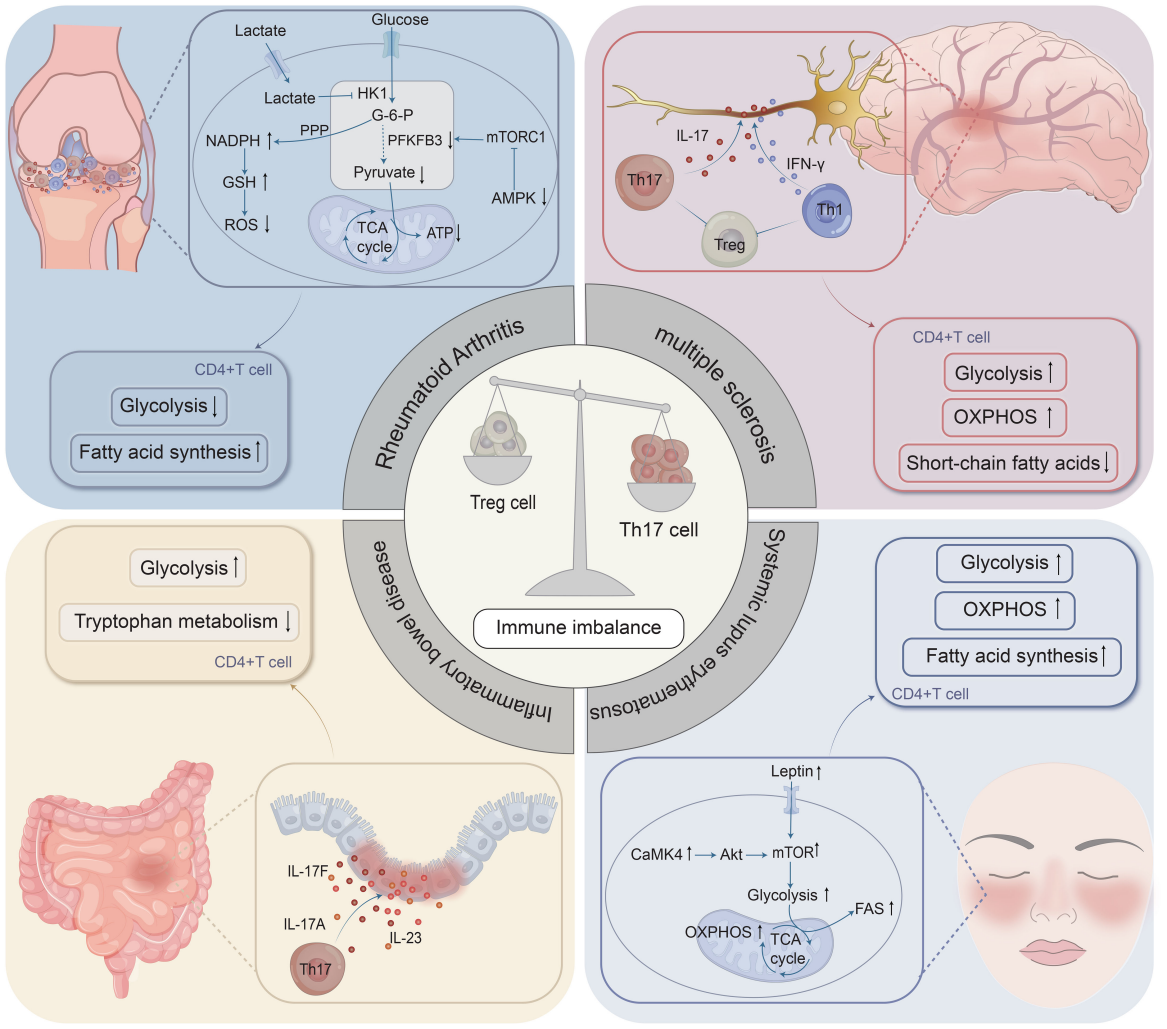
Disease-specific immunometabolic dysregulation in CD4^+^ T cells underlies several autoimmune pathologies. Rheumatoid arthritis (RA), multiple sclerosis (MS), inflammatory bowel disease (IBD), and systemic lupus erythematosus (SLE) all exhibit a Th17/Treg imbalance driven by distinct metabolic alterations. Key dysregulations include: in RA, impaired glycolytic flux and an enhanced pentose phosphate pathway in synovium; in MS, heightened glycolysis and dysregulated lipid metabolism; in IBD, defective handling of microbial metabolites like butyrate and tryptophan alongside enhanced glycolysis; and in SLE, hyperactive glycolysis, fatty acid synthesis, oxidative phosphorylation, and mTOR signaling. These shifts collectively promote pathogenic Th17 responses and compromise Treg function.

Autoimmune diseases arise from a loss of self-tolerance and aberrant immune activation, with T cells playing a central role in their pathogenesis. To systematically elucidate the critical interplay between metabolic reprogramming and T cell fate in autoimmunity, this section focuses on RA, MS, IBD, and SLE. These diseases were selected as representative models owing to distinct organ-specific manifestations and shared underlying mechanisms of immunometabolic dysregulation. Although they primarily target joints, central nervous system, gastrointestinal tract, and multiple systemic organs, respectively, a common hallmark across these diseases is a significant disruption in the balance of T cell subsets, particularly a dynamic imbalance between Th17 cells and Treg cells (**Figure 2**). The following analyses will dissect the unique and shared metabolic features that disrupt T cell homeostasis in each disease, highlighting how immunological signals and metabolic pathways converge to dictate disease progression (**Table 1**).

**Table 1:** Metabolic status in autoimmune diseases.

Disease	Pathological changes of T cell metabolism (compared with healthy people)	Parts	Clinical significance / Therapeutic implication	Reference
RA	Low glycolytic activity was displayed by CD4^+^ T cells, which switched from the glucose to the PPP.	PBMC	PPP activation in RA PBMCs suggests a metabolic adaptation for redox balance; targeting glucose fate may reprogram inflammatory T cells.	(149)
	The glycolytic pathway is impaired, and the PPP is hyperactivated in CD4^+^ T cells.	PBMC	Enhanced PPP flux in patient CD4^+^ T cells correlates with pro-inflammatory phenotype; PPP inhibition may restore metabolic and immune homeostasis.	(150)
	The glycolytic metabolism of T cells was blocked; PPP was activated and FAS increased.	PBMC	Concurrent glycolysis suppression and FAS upregulation in RA T cells highlight dual metabolic targets for intervention.	(154)
	The glycolytic activity and pyruvate level of naïve CD4^+^CD45RA^+^ T cells decreased, PPP was over-activated, and lipid synthesis increased.	PBMC and synovium	Metabolic shift in naïve T cells toward PPP and lipogenesis in early RA; suggests early metabolic intervention potential.	(295)
	The accumulation of lactic acid in synovial fluid leads to the decrease of glycolytic flux of CD4^+^T cells.	Synovium	Synovial lactate impairs T cell glycolysis and promotes IL-17 production in situ; targeting lactate transport ameliorates arthritis in murine models.	(152)
	The expression of HIF-1α and Bcl-2 increased in CD161^+^ Th cells.	Synovium	HIF-1α stabilization in synovial Th cells under hypoxia promotes pathogenic Th17 survival; HIF-1α inhibition is therapeutic in experimental arthritis.	(141)
MS	The ability of mitochondrial OXPHOS of CD4^+^T cells decreased and glycolytic metabolism increased.	PBMC	Glycolytic shift in CD4^+^ T cells from progressive MS patients; glycolytic inhibitors suppress Th17 cells in EAE models.	(172)
	Mitochondrial OXPHOS and glycolysis of CD4^+^T cells increased.	PBMC	Global metabolic activation in relapsing-remitting MS T cells; combined metabolic targeting may be required for efficacy.	(171)
	The content of propionic acid in serum and feces decreased significantly.	Serum and feces	Propionate supplementation increases Tregs and suppresses Th17 cells in MS patients; dictates microbial metabolite-based therapy potential.	(174)
	The concentration of oleic acid in adipose tissue decreased obviously.	Fat tissue	Oleic acid enhances Treg suppressive function via FAO; its deficiency in MS patients suggests dietary supplementation as a strategy.	(95)
IBD	Histidine and tryptophan decreased significantly.	Plasma	Plasma amino acid depletion in IBD patients correlates with inflammation; dietary supplementation shows efficacy in preclinical colitis models.	(205)
	The level of tryptophan in serum decreased, the level of kynurenine/tryptophan increased, and the mRNA expression of kynurenine pathway related enzymes in colon mucosa increased.	Serum and Colonic mucosa	Activated IDO-kynurenine-AhR axis in IBD mucosa promotes immune tolerance; IDO1 induction or AhR agonists show benefit in DSS-induced colitis.	(206)
	The expression of PDK4 in CD4^+^ T cells increased, and glycolytic metabolism was enhanced.	Colonic mucosa	PDK4 upregulation drives pathogenic Th17 responses in IBD mucosa; PDK4 inhibition ameliorates colitis in murine models.	(210)
	Kynurenine and kynurenine/tryptophan ratio increased significantly.	Colon tissue	Elevated kynurenine in IBD tissue signifies active IDO pathway; modulating tryptophan metabolism is a potential therapeutic avenue.	(203)
	Butyric acid decreased significantly.	Feces	Butyrate deficiency in IBD patients links dysbiosis to impaired Treg function; butyrate supplementation reduces inflammation in DSS-colitis models and enhances anti-TNF therapy response.	(196, 197)
SLE	Glycolysis metabolism and mitochondrial OXPHOS metabolism of CD4^+^ T cells increased.	PBMC	Dual metabolic hyperactivation in SLE CD4^+^ T cells drives pathogenicity; combined glycolysis and OXPHOS inhibition reverses lupus in mouse models.	(221)
	The fatty acid anabolism of Memory CD4^+^ T Cells is enhanced.	PBMC	Enhanced FAS in SLE memory T cells promotes pro-inflammatory subset expansion; ACC1 inhibition ameliorates disease in lupus-prone mice.	(231)
	Leptin levels increased significantly.	Serum and plasma	Hyperleptinemia in SLE patients promotes Th17 responses and correlates with atherosclerosis; leptin blockade improves lupus nephritis in mice.	(232, 233)
	The mTOR activity of DN T cells increased significantly, the mitochondrial membrane potential increased significantly, and the number of mitochondria increased significantly.	PBMC	mTOR hyperactivation in SLE DN T cells promotes IL-17 production; mTOR inhibition with sirolimus reduces disease activity in clinical trials.	(225)

Th17, T helper 17 cell; Treg, regulatory T cell; PPP, pentose phosphate pathway; FAS, fatty acid synthesis; OXPHOS, oxidative phosphorylation; FAO, fatty acid oxidation; HIF-1α, hypoxia-inducible factor 1-alpha; Bcl-2, B-cell lymphoma 2; PBMC, Peripheral Blood Mononuclear Cells; RA, rheumatoid arthritis; MS, multiple sclerosis; IBD, inflammatory bowel disease; SLE, systemic lupus erythematosus; EAE, experimental autoimmune encephalomyelitis; PDK, pyruvate dehydrogenase kinase 4; mTOR, mammalian target of rapamycin; DN T cells, Double Negative T cells; IDO, Indoleamine 2,3-dioxygenase; AhR, Aryl hydrocarbon receptor; DSS, dextran sulfate sodium; ACC1,acetyl-CoA carboxylase 1.

### 4.1 Rheumatoid arthritis

The pathogenic process of RA, a systemic autoimmune disease characterized by persistent synovitis, involves the activation of an aberrant immune response and the breakdown of immune system tolerance (131). In RA patients, abnormal activation and dysfunction of T cells are central to disease pathogenesis, mainly manifested by excessive activation of Th17 cell in synovial fluid and peripheral blood, along with suppression of Treg function (132). Research has revealed a notable imbalance in T cell subsets in RA patients' peripheral blood and synovium, which is typified by higher percentages of Th1 and Th17 cells and an overabundance of inflammatory cytokines (such IL-17, TNF-α, IL-23, IL-1β, etc.) (133–135). Although RA patients have abnormally high levels of Treg cells in their synovial fluid (136–140), these cells frequently have defective suppressive activity, and certain Foxp3+Treg cells may transform into pathogenic Th17 cells that secrete IL-17, thereby exacerbates inflammatory responses (141). The Th17/Treg balance significantly correlates with disease activity in RA patients (142). However, conflicting data exist regarding Treg frequency and function in peripheral blood mononuclear cells (PBMCs) of RA patients (143), with reports of increased (144, 145), normal (136, 137) or reduced (138, 139, 146, 147) numbers, potentially reflecting individual variations and disease activity status. Significantly, the percentage of follicular regulatory T cells (Tfr) in peripheral blood increases as RA patients achieve remission; this increase is inversely correlated with serum autoantibody levels, indicating that Tfr cells may be protective in controlling autoantibody production and preserving immunological homeostasis (148).

Metabolically, T cells from RA patients exhibit reduced glycolytic activity and compromised mitochondrial function (**Figure 2**). CD4^+^ T cells from RA patients demonstrate decreased expression of the glycolytic rate-limiting enzyme PFKFB3 compared with healthy donor PBMCs, resulting in diminished pyruvate and ATP production (149). Concomitant shunting of glucose metabolism toward the pentose phosphate pathway increases NADPH production for reactive oxygen species (ROS) scavenging, enhancing cellular susceptibility to apoptosis (149). Conversely, naive CD4^+^ T cells, exhibit increased production of glucose-6-phosphate dehydrogenase (G6PD), which stimulates Th1 and Th17 cell development and intensifies inflammatory responses (150). In local synovial tissues, the hypoxic environment favors stable expression of HIF-1α, enhancing the survival of pathogenic CD4^+^ T cell and further aggravating inflammation (14, 141, 151). By activating the lactate transporter Slc5a12 on CD4^+^ T cells and inhibiting the expression of hexokinase 1 (HK1), lactate accumulation in synovial fluid (SF) reduced glycolytic flux in T cells and promotes FAS, which raises IL-17 production (152).

It has been discovered that AMPK activation may have therapeutic benefits in reducing RA inflammation. In murine arthritis models, metformin reduces arthritic symptoms by blocking Th17 cell development through the AMPK-mTOR pathway (153). However, functional defects in N-myristoyltransferase (NMT) within T cells of RA patients impede AMPK activation, leading to hyperactivation of the mTORC1 pathway and encourages the proliferation of Th1 and Th17 cells (154). This condition can be reversed by pharmacologically activating AMPK or overexpressing NMT1, which will restore metabolic balance (154).

In addition, activated B cells in RA patient peripheral blood drive inflammatory T helper cell activation and cytokine production via the ICOS-ICOSL pathway, while concomitantly upregulating T cell Glut1 expression to enhance glucose uptake (155). The observed reduction in T cell Glut1 levels following rituximab-induced disease remission underscores the significance of T-B cell metabolic crosstalk in RA pathogenesis.

Taken together, current evidence indicates that T cells in RA patients exhibit a distinct metabolic signature characterized by dysregulated glycolysis and impaired mitochondrial function. This metabolic reprogramming promotes Th17/Treg imbalance and sustains synovial inflammation, underscoring the therapeutic potential of AMPK activation and metabolic modulation.

### 4.2 Multiple sclerosis

MS is a chronic autoimmune disorder of the central nervous system (CNS) characterized by neuroinflammation and neurodegenerative changes (156, 157). Its pathogenesis primarily involves immune dysregulation, including breakdown of immune tolerance, alterations in the immune microenvironment, and dysregulation of T cell subsets (158, 159).

In MS, Th1 and Th17 cells promote neuroinflammation and demyelination through secretion of pro-inflammatory cytokines such as IFN-γ and IL-17 (160, 161). Treg frequency in peripheral blood is not consistently reduced, their suppressive function is significantly impaired, a key factor in disease progression (162–166). Restoration of Treg cell function (167) or inhibition of the pro-inflammatory activity of Th cells (168) can effectively alleviate MS symptoms. Teff in MS patients' peripheral blood exhibit particular resistance to Treg cell-mediated suppression, further exacerbating immune imbalance (162, 169, 170).

Metabolically, T cells in MS display dynamic and context-dependent alterations. Studies have revealed metabolic heterogeneity across different disease phases and clinical subtypes. For instance, Klotz et al. reported that during active relapse in relapsing-remitting MS (RRMS), T cells exhibit elevated OXPHOS and glycolysis compared to healthy controls (171). This hypermetabolic state may reflect heightened activation and proliferation of autoreactive T cell clones. In contrast, De Biasi et al. observed reduced OXPHOS and a shift toward glycolysis in CD4^+^ T cells from patients with progressive MS, particularly in the primary progressive (PP) form (172). This metabolic profile was associated with mitochondrial dysfunction, lower mitochondrial mass, and increased expression of glycolytic genes, suggesting a distinct immunometabolic phenotype in progressive disease stages.

Notably, CD4^+^ T cells from PPMS patients showed a stronger glycolytic response upon activation compared to those from secondary progressive (SPMS) patients (172), potentially indicating a more severe or chronic inflammatory state in the PP subtype. These discrepancies highlight the importance of considering disease phase (relapsing or progressive), clinical subtype (RRMS, PPMS, SPMS), and activation status when interpreting T cell metabolism in MS.

Targeting metabolic pathways represents a promising therapeutic strategy. The microbial metabolite itaconate reduces Th17 differentiation and promotes Treg generation by inhibiting both glycolysis and OXPHOS (173). In EAE mice, adoptive transfer of itaconate-treated, Th17-polarized T cells significantly ameliorated disease severity (173), highlighting the potential of targeting metabolic pathways to modulate the Th17/Treg balance in autoimmune neuroinflammation.

Clinical evidence indicates reduced propionate levels in the serum and feces of patients with MS (174). Propionate supplementation increases Treg frequency and function while decreasing Th1 and Th17 cells in human (174). Similarly, oleic acid supplementation can partially restore the suppressive function of Treg cell by improving FAO-driven OXPHOS metabolism, thereby enhancing the expression of Foxp3 and STAT5 phosphorylation in MS patients (95). In animal models, the therapeutic potential of propionate has been corroborated in EAE (175). Propionate treatment was found to ameliorate the aggravated clinical course of EAE induced by a high-fat diet (HFD) rich in lauric acid. This was associated with reduced demyelination and immune cell infiltration in the spinal cord. Mechanistically, propionate rescued HFD-enhanced immunopathology by inhibiting pro-inflammatory Th17 responses and increasing the frequency and functionality of Tregs. These beneficial effects were linked to the suppression of p38-MAPK phosphorylation and a dependence on IL-10 signaling (175).

Notably, the association between obesity and MS risk is supported by causal evidence from Mendelian randomization studies. These studies demonstrate that a genetically determined higher body mass index (BMI) is causally associated with an increased susceptibility to MS, with this effect being observed both in the adult population (176) and, more specifically, in pediatric-onset disease (177). These findings underscore substantial disruptions in lipid metabolism in MS and highlight the role of various fatty acids in modulating Treg cell function and regulating the inflammatory milieu in this disease.

In conclusion, MS involves complex metabolic alterations in T cells, with enhanced glycolysis and disrupted lipid metabolism contributing to Th17/Treg imbalance, while microbial metabolites and fatty acids offer promising therapeutic avenues for immune resetting.

### 4.3 Inflammatory bowel disease

IBD is a group of disorders characterized by chronic recurrent gastrointestinal inflammation, primarily including Crohn's Disease (CD) and Ulcerative Colitis (UC). Although the etiology of IBD remains unclear, it is generally considered to be the result of a confluence of environmental factors (such as nutrition and microbiota), immune system dysregulation, intestinal epithelial barrier impairment, and genetic predisposition (178). Recent research has demonstrated that the pathophysiology of IBD is largely influenced by T cell-mediated immune responses in the intestinal mucosa, specifically the imbalance in the dynamic equilibrium between Th17 and Treg cells, which is crucial for the onset and progression of IBD (179).

Intestinal tissues of IBD patients show elevated Th17 cells and pro-inflammatory factors (IL-17A, IL-17F, IL-23), contributing to intestinal inflammation (180–182). Nevertheless, the role of IL-17A in IBD is complicated. Though IL-17A is typically thought of as a pro-inflammatory factor, its absence actually exacerbates intestinal inflammation in CD and compromises the barrier function of intestinal epithelial cells, whereas inhibiting IL-23 secretion significantly reduces inflammatory responses (183–185). This may be due to the protective role of IL-17A in the intestines (186). Genome-wide association studies (GWAS) further revealed that genes related to Th17 cell (such as STAT3, IL-12B) are closely associated with CD susceptibility (187). Treg cells are abundant in the peripheral blood and intestines of IBD patients (188). This could be the body's compensatory reaction to inflammation, but their function is frequently compromised, making it difficult for them to effectively suppress inflammatory responses. Thus, targeting Th17 cells and their associated pathways has thus emerged as a key therapy strategy for IBD (189–192), with metabolic reprogramming of the Th17/Treg balance holding significant promise.

The primary mechanism for the chronic progression of IBD in individuals is localized immune-metabolic imbalance in the gut (**Figure 2**). Notably, intestinal tryptophan metabolism shortcomings (AhR signaling deficiencies) and poor butyrate consumption are especially important. In addition to providing colonic epithelial cells with an energy substrate to sustain barrier function, butyrate also promotes Treg cell development via HDAC inhibition–mediated histone H3K27 acetylation at the Foxp3 gene locus (193–195). Clinical metabolomics analyses reveal that IBD patients have lower butyrate concentrations and a much lower number of butyrate-producing bacteria (Faecalibacterium prausnitzii and Roseburia hominis) than healthy controls (196–198). Butyrate supplementation successfully reduces intestinal inflammatory damage in DSS-induced murine colitis (199, 200). Notably, baseline levels of butyrate and butyrate-producing species positively correlate with clinical response to anti–TNF-α therapy in humans (201, 202), highlighting butyrate's role in restoring epithelial barrier function, immune regulation, and biologic therapy efficacy.

Tryptophan metabolism is also disrupted in IBD. Patients show significantly lower tryptophan levels in serum and feces, with an increased kynurenine/tryptophan ratio indicating enhanced IDO1 activity (203–206). Dietary L-tryptophan supplementation demonstrates stage-specific immunomodulatory effects; it increases colonic HELIOS+ Tregs via the AhR-GPR15 pathway and prevents DSS-induced colitis in mice when administered prophylactically, but shows limited efficacy after epithelial barrier disruption (207).

Moreover, increased glycolysis capability is directly linked to excessive Th17 cell activation (66). While dietary restriction can reduce inflammation, high-fat and high-sugar diets raise the incidence of IBD (208, 209) and may worsen intestinal inflammation by encouraging a Th17/Treg imbalance (179). In colon biopsy samples from IBD patients, CD4^+^ T cells show markedly increased pyruvate dehydrogenase kinase (PDK) expression, and PDK4 inhibition alleviates intestinal inflammation in a DSS-induced murine colitis model (210). Further demonstrating the therapeutic potential of targeting glycolysis, the natural compound derivative D5, sourced from Radix Aucklandiae, attenuates colitis in mice by impeding the nuclear translocation of PKM2 dimers, thereby suppressing glycolytic flux and Th17 cell differentiation (211).

Therefore, localized immunometabolic disturbances, particularly defective butyrate metabolism and dysregulated tryptophan catabolism, disrupt the Th17/Treg balance and compromise epithelial integrity in IBD, underscoring the gut microbiome and microbial metabolites as actionable therapeutic targets.

### 4.4 Systemic lupus erythematosus

SLE is a systemic autoimmune disease that is driven by environmental variables as well as hereditary susceptibility. It is characterized by immune system dysregulation that causes inflammation and damage multiple organs (212). Its pathophysiology is primarily caused by uncontrolled immune system activation, specifically T cell and B cell malfunction, which leads to an overabundance of autoantibodies and immune complex deposition, both of which cause tissue damage. Research suggests that the pathogenic mechanism of SLE is significantly influenced by the imbalance between Th17 and Treg cells (213–217). Patients with SLE exhibit decreased Treg cells and compromised function in alongside a higher proportion of Th17 cells, which is positively correlated with the severity and advancement of the disease (218, 219).

T cell metabolic dysregulation profoundly influences SLE pathophysiology (220). Research has demonstrated that CD4^+^ T cells in lupus-prone mice and SLE patients displayed greater mitochondrial dysfunction and glycolysis metabolism (**Figure 2**). In addition to encouraging Th17 cell differentiation, this metabolic anomaly inhibits Treg cell function, resulting in a Th17/Treg imbalance and aggravating the course of the disease (221–223). Unlike some other autoimmune conditions, SLE T cells exhibit hyperactivation of the mTOR signaling pathway. In particular, mTORC1 activity is markedly elevated in SLE patients' T cells, which stimulates the production of IL-4 and IL-17 (224), while inhibiting Foxp3 expression, which reduces the immune-suppressive capacity of Treg cells (225). Crucially, medications like Rapamycin that target the mTOR signaling system have shown great promise as treatments. By blocking the activity of mTORC1, research shows that Rapamycin decreases the production of IL-4 and IL-17, encourages the growth of Treg cells, and ultimately decreases the activity of SLE disease (224, 226). Moreover, a multifunctional serine/threonine kinase called calcium/calmodulin-dependent protein kinase IV (CaMK4) plays an essential role in SLE and promotes the differentiation and activity of Th17 cells. T cells from SLE patients (227, 228) and lupus-prone mice (229) have abnormally high expression of CaMK4, which activates the AKT-mTOR pathway to promote Th17 cell development and IL-17 release.Th17 cell differentiation can be considerably decreased by blocking the CaMK4-AKT-mTOR pathway (230). However, there aren't many proven CaMK4 inhibitors available for the clinical management of SLE at the moment. In order to give SLE patients additional treatment options, future research must investigate its regulatory processes in greater detail and create safer and more potent inhibitors.

The pathogenic mechanisms of SLE, which encourage Th1 cell differentiation and excessive production of IFN-γ and result in treatment resistance, are likewise associated with the enhancement of FAS (231). Because it inhibits the proliferation of Treg cells, leptin, a pro-inflammatory adipocytokine, is unusually increased in SLE patients and speeds up the course of lupus nephritis (232, 233). It's interesting to observe that in lupus models, autoantibody synthesis and immunological imbalance are tightly linked to abnormal leptin expression. By blocking the leptin signaling pathway, it is possible to efficiently restore the number and function of Treg cells, which would slow the progression of the disease (234). Leptin is a key pro-inflammatory element in the pathogenic phase of SLE, as established by these processes taken together. Surprisingly in female SLE patients, elevated leptin levels are independently correlated with a higher risk of atherosclerosis (233). Leptin-targeting anti-leptin treatments are still in the research and exploratory stage, however, and more empirical and theoretical evidence based on the leptin signaling pathway is needed to develop novel therapeutic approaches for clinical metabolic or immunological diseases.

Collectively, these findings demonstrate that SLE is characterized by mTOR pathway hyperactivation and distinct T cell metabolic reprogramming favoring glycolysis and FAS, which disrupts Th17/Treg balance and drives autoimmunity, positioning metabolic intervention as a viable treatment strategy.

These findings demonstrate profound interconnections between metabolic dysfunction and immune dysregulation across RA, MS, IBD, and SLE. While metabolic biases differ—impaired glycolysis in RA T cells versus enhanced glycolysis in MS and SLE—they converge on a common immunological phenotype: Th17 cell hyperactivation and Treg cell functional impairment (**Figure 2**). Tissue-specific metabolites exert powerful immunomodulatory effects: lactate in RA synovium reinforces Th17 pathogenicity; propionate and oleate in MS enhance Treg function via FAO; and butyrate in IBD maintains mucosal Treg populations and epithelial integrity. The HIF-1α pathway is frequently activated in hypoxic inflammatory sites (RA joint, IBD gut), stabilizing Th17 responses and antagonizing Treg stability. Correcting this imbalance through targeted inhibition of pro-inflammatory pathways or supplementation of regulatory metabolites can reset immunological homeostasis, underscoring the translational potential of metabolic checkpoint therapy in autoimmunity.

## 5 Metabolic targeting therapeutic strategies

**Figure 3:**
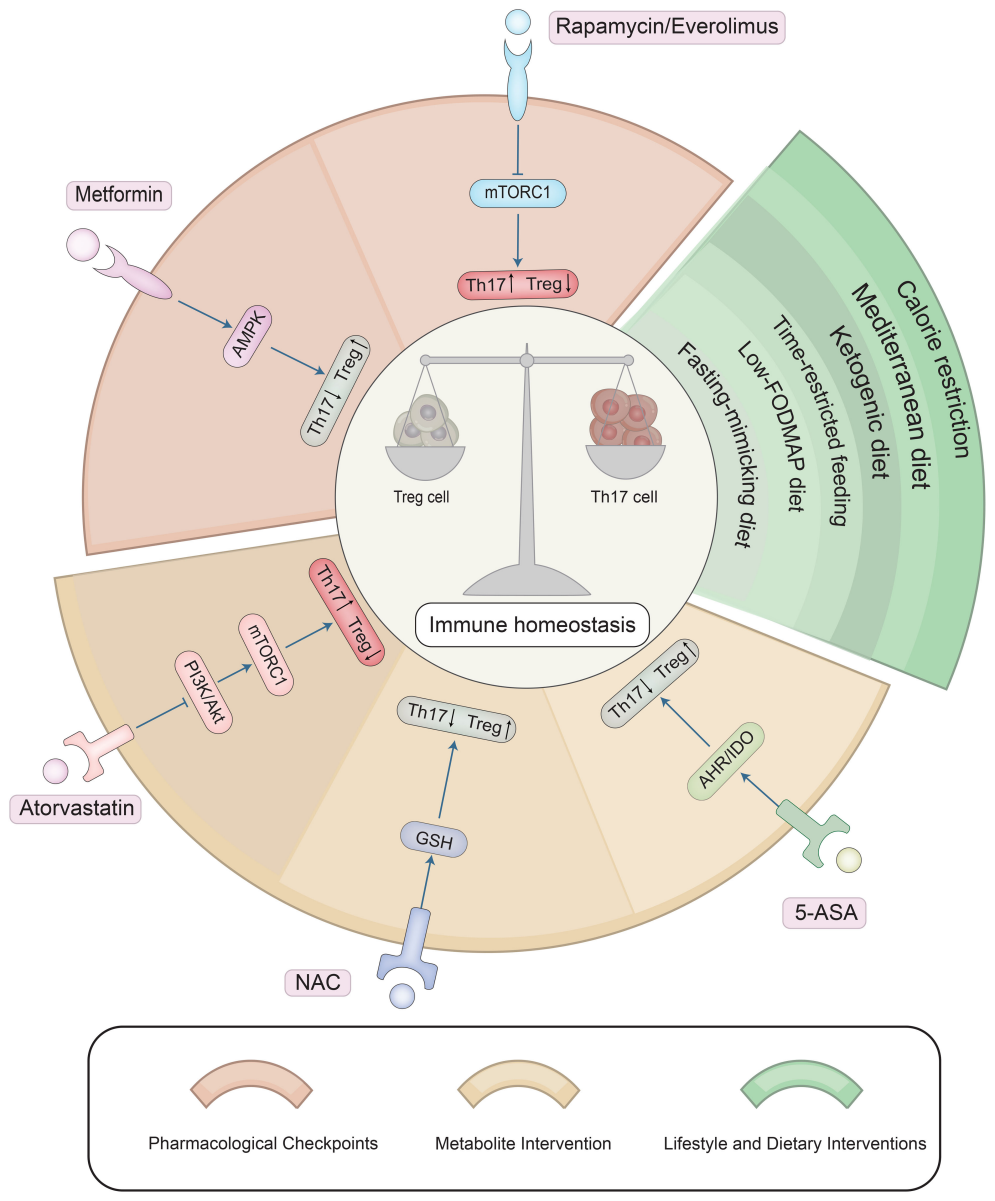
Therapeutic strategies targeting metabolic pathways to restore the Th17/Treg balance. Core pharmacological interventions include: mTOR inhibitors (Rapamycin) to suppress glycolysis and promote Tregs; AMPK activators (Metformin) to enhance fatty acid oxidation; NAC and 5-ASA to modulate oxidative stress and kynurenine pathways; Statins (Atorvastatin) to inhibit PI3K-Akt-mTOR signaling. Dietary and lifestyle approaches—such as caloric restriction, ketogenic diets, and butyrate supplementation—are shown as complementary strategies to support metabolic balance and regulatory immunity.

Targeting T cell metabolic reprogramming as a therapeutic strategy has advanced significantly in the fields of immunotherapy and autoimmune diseases in recent years. By controlling T cell metabolic pathways like glycolysis, OXPHOS, FAO, and amino acid metabolism, these tactics may effectively regulate T cell differentiation, function, and immune response, offering a fresh approach to the treatment of autoimmune diseases (**Figure 3**). The medications that currently target T cell metabolism in clinical treatment are compiled in the following (**Table 2**).

**Table 2:** Clinical drugs related to metabolism.

Medicine	Target	Metabolic pathway	Autoimmune diseases treated	Stage of development	Clinicaltrials.gov/ Chictr.org.cn identifier	Reference
Sirolimus/ Everolimus	mTORC1/2	Glycolysis	RA, SLE	Approved (transplant); Phase I/II in SLE & RA	NCT00779194 (USA) ChiCTR-IPR-17010307 (China)	(142, 236, 240, 244, 245)
Metformin	AMPK	Glycolysis, Lipid metabolism	RA, SLE	Approved (T2D); Phase II/III in SLE & RA	NCT02741960 (China) NCT04068246 (Egypt)	(249–252, 296)
NAC	GSH	Glutamate metabolism	SLE	Phase II (RCT completed) in SLE	NCT00775476 (USA)	(253–255)
5-ASA	AhR-IDO1	Tryptophan metabolism	UC	Approved (UC)	NCT00505778 (USA)	(259, 260, 297)
Atorvastatin	PI3K-Akt-mTOR and ERK signals	Lipid metabolism	Adjuvant Treatment of SLE, MS, RA and other diseases	Approved (dyslipidemia); Phase II/III in autoimmune settings	NCT00942591 (Switzerland)	(274–278, 280, 282)

mTORC1/2, mammalian target of rapamycin complex 1/2; AMPK, AMP-activated protein kinase; PI3K, phosphoinositide 3-kinase; UC, Ulcerative Colitis; RA, rheumatoid arthritis; MS, multiple sclerosis; SLE, systemic lupus erythematosus; AhR, Aryl hydrocarbon receptor; IDO1, Indoleamine 2,3-dioxygenase 1; T2D, Type 2 Diabetes Mellitus; RCT, Randomized Controlled Trial; NAC, N-acetylcysteine; 5-ASA, 5-aminosalicylic acid (Mesalazine); Akt, Protein Kinase B; ERK, Extracellular Signal-Regulated Kinase.

### 5.1 Pharmacological checkpoints

#### 5.1.1 mTOR inhibitors

The mTOR pathway is a central regulator of cellular metabolism and growth. Sirolimus (rapamycin), a first-generation mTORC1 inhibitor approved for transplant rejection, has demonstrated efficacy in autoimmunity (235). In a 24-week open-label Phase I/II clinical trial (Clinicaltrials.gov Identifier: NCT00779194) in patients with active SLE, sirolimus treatment increased the proportion of circulating Foxp3+ Tregs, suppressed IL-17 and IL-4 secretion, and facilitated glucocorticoid tapering (mean prednisolone dose reduced from 23.7 mg to 7.2 mg daily) (236). Notably, Through a feedback mechanism, mTORC1 inhibition may momentarily increase mTORC2 signaling, causing temporary metabolic disruptions during the first phase of treatment (224), which can transiently disrupt metabolism and frequently leads to hyperlipidemia, necessitating concomitant lipid-lowering therapy (237–239). The mechanism may be caused by aberrant hepatic lipid-producing pathways and mTORC2-mediated transcriptional activation of SREBP-1c, underscoring the need for combined lipid-lowering therapy. In patients with active RA, the addition of low-dose sirolimus (0.5 mg every other day for 24 weeks) to conventional immunosuppressive therapy effectively increased Treg cell frequencies, reduced Th17 cell populations, and improved clinical disease activity (Chictr.org.cn identifier: ChiCTR-IPR-17010307) (240). This combination regimen demonstrated a superior safety profile compared to immunosuppressant monotherapy, while mitigating risks of overtreatment-related adverse events such as hepatotoxicity and serious infections (240). Notably, in RA patients with low disease activity, a 12-week course of low-dose sirolimus (0.5 mg every other day) also exhibited a favorable safety profile, elevating Treg levels and enhancing clinical outcomes without evidence of toxicities—such as hemorrhagic cystitis or liver fibrosis—that are associated with certain conventional immunosuppressive agents (142). These findings collectively suggest that low-dose sirolimus possesses a favorable risk-benefit ratio in RA patients across varying disease activity states, supporting its potential as an immunomodulatory adjunct in RA management.

Everolimus, a second-generation mTOR inhibitor, modulates T cell proliferation, metabolism, and apoptosis by specifically inhibiting the mTORC1/2 complex (241–243). A small trial in methotrexate (MTX)-resistant RA suggested that everolimus plus MTX could alleviate joint symptoms, though larger confirmatory studies are needed (244). Its efficacy appears disease-specific. A case report described a 26-year-old female with SLE and tuberous sclerosis complex (TSC) whose nephrotic syndrome was controlled, and corticosteroid dose was steadily reduced during 6 years of everolimus treatment, highlighting its potential in overlapping pathologies (245). Conversely, a clinical trial demonstrated that everolimus was no more effective than placebo for the maintenance of remission in CD (246).

#### 5.1.2 AMPK activators

Metformin, a first-line antidiabetic drug and AMPK agonist, exerts immunometabolic effects primarily by restraining mTOR signaling (247). Mechanistically, it inhibits glycolysis and mitochondrial hyperactivation in CD4^+^ T cells, thereby suppressing Th1/Th17 cell differentiation and promoting Treg cell expansion (221, 248).

Clinical trials across autoimmune diseases indicate a potential role for metformin as an adjunct therapy. In a 12-week, randomized, double-blind, placebo-controlled trial (RCT) in active RA (Clinicaltrials.gov: NCT04068246, n=120) demonstrated that adjunct metformin (1000 mg/day) to methotrexate (MTX; 7.5 mg) significantly improved clinical outcomes (249). The primary endpoint, ACR20 response rate, was 80.8% in the metformin group versus 54.7% in the placebo group (P = 0.001). Significant improvements were also observed in key secondary endpoints including ACR50, ACR70, and DAS28-3 (CRP) (249). A 12-month RCT (Clinicaltrials.gov: NCT02741960; n=140) and a preceding proof-of-concept, open-label study (n=113) collectively indicated that metformin can reduce prednisone exposure and may lower the risk of disease relapse, particularly major flares (250, 251). The primary efficacy endpoint of the larger trial did not reach statistical significance, warranting confirmation in future studies. Both studies confirmed a well-tolerated safety profile for metformin. For both RA and SLE trials, the safety profile was favorable, with no serious adverse events attributed to metformin. The most frequent adverse events were gastrointestinal (e.g., RA: nausea 10% vs. 8.33%; SLE: 39% vs. 15%), which were typically mild and manageable (249, 251). In a rat model of adjuvant-induced arthritis, the combination of metformin (200 mg/kg/day) and omega-3 (300 mg/kg/day) showed efficacy comparable to methotrexate (2 mg/kg/week) in ameliorating disease activity, with significant reductions in arthritic scores, paw swelling, and serum TNF-α and IL-1β after 4 weeks (252). This combination also conferred hepatoprotection by normalizing transaminase levels. Although gastrointestinal tolerability was not explicitly evaluated, metformin is associated with such adverse effects in clinical settings, a factor relevant for translational consideration.

### 5.2 Metabolite intervention

#### 5.2.1 N-acetylcysteine

N-Acetylcysteine (NAC), as a classic antioxidant, has shown potential as a therapeutic option for systemic autoimmune diseases in recent years. As shown by studies, NAC not only enhances GSH by supplying cysteine precursors, reverses the oxidative stress state in SLE patients' T cells, and counteracts GSH depletion brought on by mitochondrial dysfunction in SLE patients' T cells (253). It also lowers Kyn levels by preventing IDO activity, which prevents mTOR activation and lessens disease activity in SLE patients (254). A RCT verified that patients treated with NAC had higher GSH levels in PBMCs, along with upregulated Foxp3 expression and suppressed mTOR activity (lower expression of phosphorylated S6 protein), which ultimately resulted in lower anti-dsDNA antibody titers and SLEDAI scores (Clinicaltrials.gov: NCT00775476) (255).

#### 5.2.2 Mesalazine

Mesalazine (5-ASA) is a first-line therapeutic agent and standard of care for UC. Beyond its well-established local anti-inflammatory actions, emerging evidence suggests that 5-ASA contributes to mucosal immune homeostasis through immunometabolic regulation—not as a T cell–specific drug, but via broader mechanisms including activation of the AhR–IDO1 axis (256). This pathway promotes tryptophan conversion to kynurenine, enhances Treg cell populations within the intestinal lamina propria, and suppresses IL-17A production (256–258).

Clinical meta-analyses support individualized 5-ASA dosing strategies based on disease severity (259, 260). For patients with mild to moderate (grade I–II) UC, particularly those with left-sided or rectal involvement, topical 5-ASA is often recommended. Those with moderately active disease may benefit from high-dose oral 5-ASA (>3 g/day), while combined oral and topical 5-ASA can improve remission rates in patients with mild active or extensive UC who respond inadequately to oral monotherapy. Thus, 5-ASA administration should be tailored according to disease extent, severity, and individual patient factors.

### 5.3 Combination therapies

#### 5.3.1 Low-dose IL-2

It is well-established that IL-2 plays a pivotal role in T cell development, demonstrating a preferential capacity to promote the expansion and functional specialization of Treg, Th1, Th2, and Th9 cells, while it does not directly support the differentiation of Th17 and T follicular helper (Tfh) cells (261, 262). In autoimmune conditions, including RA and SLE, IL-2 production is significantly diminished, which contributes to the characteristic decline in Treg cell frequency and functional impairment, thereby disrupting immune tolerance (263–265). therapy has been developed as a targeted immunomodulatory strategy to specifically correct this Treg cell defect. Clinical trials across various autoimmune diseases, such as SLE, RA, type 1 diabetes, and Primary Sjögren's Syndrome, have demonstrated that Low-dose IL-2 can safely and effectively induce stable Treg cell proliferation and restore immune balance, leading to improved disease activity (266–271).

Notably, combination therapies integrating Low-dose IL-2 with metabolic immunomodulators can further enhance the stability and function of Treg cells. In a clinical study involving patients with refractory SLE—defined as those with persistent disease activity despite high-dose glucocorticoids and/or cytotoxic drugs—the combination of Low-dose IL-2 (100 WIU, 3–5 days/month) and rapamycin(0.5 mg every other day) demonstrated synergistic effects (272). This regimen significantly increased Treg cell numbers at 12 and 24 weeks, restored the Th17/Treg balance, and reduced disease activity as measured by SLEDAI scores (272). Importantly, the therapy facilitated a substantial reduction in prednisone dosage at 6, 12, and 24 weeks, underscoring its steroid-sparing potential. No serious adverse events were reported, indicating a favorable safety profile for this combination approach (272).

#### 5.3.2 Statins

Statins are essential medications for the treatment of hyperlipidemia and atherosclerotic cardiovascular disorders because they are 3-hydroxy-3-methylglutaryl coenzyme-A (HMG-CoA) reductase inhibitors, which can dramatically lower low-density lipoprotein cholesterol (LDL-C) levels (273). Particularly, atorvastatin can be used as an adjuvant treatment in addition to decreasing cholesterol. It considerably reduces cardiovascular events and improves inflammatory state and disease activity scores when used in conjunction with biologics for autoimmune diseases (such as RA and SLE) (274–277). This might have to do with its capacity to prevent the PI3K-Akt-mTOR and Extracellular Signal-Regulated Kinase (ERK) pathways, which enhances suppressive functioning and increases the number of Treg cells (278).

However, atorvastatin may have dose-dependent effects when used as an adjuvant treatment for multiple sclerosis. Following early research, adding high-dose atorvastatin to individuals with stable relapsing-remitting multiple sclerosis receiving high-dose IFN-β 1a increased disease activity (279). Conversely, patients who did not respond well to high-dose IFN-β 1a monotherapy seemed to benefit more from low-dose atorvastatin (280). IFN-β 1b plus atorvastatin has been shown in some studies to significantly lower high-sensitivity C-reactive protein levels in patients, indicating an anti-inflammatory effect of atorvastatin in MS (281). However, an RCT (n=77) revealed that in relapsing-remitting MS patients, IFN-β 1b plus atorvastatin was more likely to cause temporary elevations in liver enzymes than monotherapy, and the combined therapy was not better than monotherapy (Clinicaltrials.gov: NCT00942591) (282). It's interesting to note that it can considerably prolong the relapse time in MS patients by lowering the levels of pro-inflammatory factor IFN-γ and raising the levels of anti-inflammatory factors IL-35 and IL-10 when paired with methylprednisolone (283). These results imply that atorvastatin, when used as a supplemental treatment for autoimmune diseases, necessitates careful consideration of dosage and combination medication specificity, as well as caution against drug-induced liver damage from interactions.

### 5.4 Lifestyle and dietary interventions

Specifically, adjusting food patterns, enhancing habits, and taking essential nutrients as supplements can help avoid autoimmune diseases to some extent in addition to reducing their symptoms. As an example, it has been discovered that calorie restriction (CR) and the ketogenic diet (KD) have beneficial benefits on MS via a variety of pathways. In particular, these dietary patterns can inhibit Th17 cell differentiation, increase Treg cell proliferation, and significantly lower the expression levels of pro-inflammatory eicosanoid biosynthesis enzymes (like COX1, COX2, and ALOX5), all of which can mitigate inflammatory responses in MS patients and enhance their quality of life (284–286). Additionally, research in the EAE model has found that KD can inhibit the GSDMD and JAK2-STAT3/4 pathways, decrease T cell migration and infiltration into the CNS, promote Treg cell differentiation, and decrease Th17 cell differentiation in the spleen and spinal cord (287), providing new therapeutic approaches for MS.

Individual reactions to certain diets differ, as several studies have proven (288–290). For example, the Mediterranean diet (MD), which is high in dietary fiber, omega-3 fatty acids, and polyphenols, can help IBD patients achieve clinical remission and improve their nutritional status. The low-FODMAP diet (LFD), on the other hand, limits fermentable short-chain carbohydrates, which lowers intestinal gas production and inflammatory responses. Time-restricted feeding (TRF) has been discovered to decrease intestinal inflammation in DSS-induced colitis models by increasing the number of CD4^+^CD25^+^ T cells and decreasing the fraction of CD4^+^ T cells in peripheral blood and mesenteric lymph nodes (291). By lowering intestinal inflammation, boosting stem cell counts, and encouraging the development of protective microbiota, the fasting-mimicking diet (FMD) dramatically improves DSS-induced colitis models (292, 293). Importantly, targeted delivery of metabolites like butyrate can more successfully restore intestinal homeostasis and reduce inflammatory responses (294). IBD patients frequently have gut dysbiosis, including a decrease in butyrate-producing bacteria (197). Therefore, one of the most important future directions for the treatment of IBD is the formulation of personalized diet plans based on individuals' immunometabolic status and microbiota profiles.

## 6 Conclusions

This review systematically delineates the pivotal metabolic axes that govern the Th17/Treg balance in autoimmune diseases, with a focus on RA, SLE, MS, and IBD. The distinct metabolic preferences of Th17 (glycolysis and FAS) and Treg cells (OXPHOS and FAO), underscore the potential of metabolic reprogramming as a therapeutic strategy. Shifting T cell metabolism from a hyper-glycolytic state toward OXPHOS and FAO represents a promising approach to restore immune homeostasis.

Preclinical studies in animal models, such as EAE and DSS-induced colitis, have consistently demonstrated the efficacy of targeting metabolic nodes like mTOR, AMPK, HIF-1α, and PKM2 in rebalancing Th17/Treg responses. However, the translation of these findings into human applications remains challenging. While mTOR inhibitors (e.g., sirolimus) and AMPK activators (e.g., metformin) have shown promise in clinical trials for SLE and RA, their effects are often accompanied by off-target consequences, such as hyperlipidemia and infection risk, highlighting the need for more selective targeting. Conversely, interventions like Low-dose IL-2 and NAC have demonstrated encouraging immunomodulatory effects in human trials, though their long-term efficacy and safety require further validation.

Notably, several promising approaches remain largely confined to preclinical stages. For instance, TEPP-46 and microbial metabolite-based therapies (e.g., butyrate, itaconate) exhibit robust efficacy in animal models but have yet to be systematically evaluated in human autoimmune populations. Similarly, dietary interventions such as ketogenic and fasting-mimicking diets show metabolic and immunologic benefits in mice, but their applicability and sustainability in human patients warrant rigorous clinical assessment.

Future efforts should prioritize the identification of predictive metabolic biomarkers through integrated multi-omics and single-cell technologies, enabling patient stratification for personalized metabolic therapy. Moreover, advanced delivery systems—such as nanoparticle-based targeting and gut microbiota engineering—may enhance the specificity and efficacy of metabolic immunomodulators while minimizing systemic toxicity. By bridging the gap between preclinical insight and clinical validation, we can advance toward a new era of precision immunometabolism therapy, ultimately improving long-term outcomes for patients with autoimmune diseases.
